# Hippocampal–anterior thalamic pathways for memory: uncovering a network of direct and indirect actions

**DOI:** 10.1111/j.1460-9568.2010.07251.x

**Published:** 2010-06

**Authors:** John P Aggleton, Shane M O’Mara, Seralynne D Vann, Nick F Wright, Marian Tsanov, Jonathan T Erichsen

**Affiliations:** 1 School of Psychology, Cardiff UniversityTower Building, Park Place, Cardiff, Wales CF10 3AT, UK; 2 Institute of Neuroscience and School of Psychology, Trinity CollegeDublin, Ireland; 3 School of Optometry and Vision Sciences, Cardiff UniversityCardiff, UK

**Keywords:** amnesia, fornix, hippocampus, mammillary bodies, recognition memory, spatial memory, thalamus

## Abstract

This review charts recent advances from a variety of disciplines that create a new perspective on why the multiple hippocampal–anterior thalamic interconnections are together vital for human episodic memory and rodent event memory. Evidence has emerged for the existence of a series of parallel temporal–diencephalic pathways that function in a reciprocal manner, both directly and indirectly, between the hippocampal formation and the anterior thalamic nuclei. These extended pathways also involve the mammillary bodies, the retrosplenial cortex and parts of the prefrontal cortex. Recent neuropsychological findings reveal the disproportionate importance of these hippocampal–anterior thalamic systems for recollective rather than familiarity-based recognition, while anatomical studies highlight the precise manner in which information streams are kept separate but can also converge at key points within these pathways. These latter findings are developed further by electrophysiological stimulation studies showing how the properties of the direct hippocampal–anterior thalamic projections are often opposed by the indirect hippocampal projections via the mammillary bodies to the thalamus. Just as these hippocampal–anterior thalamic interactions reflect an interdependent system, so it is also the case that pathology in one of the component sites within this system can induce dysfunctional changes to distal sites both directly and indirectly across the system. Such distal effects challenge more traditional views of neuropathology as they reveal how extensive covert pathology might accompany localised overt pathology, and so impair memory.

## Introduction

While the connections of the hippocampus are surely vital for memory, determining which of its many connections are particularly important and why they are important remain outstanding problems. It is only by answering these questions that we can ever develop a comprehensive model of medial temporal lobe memory mechanisms. This review begins by considering the importance of the fornix, the largest single pathway linking the hippocampus with distal brain sites. The first section highlights recent neuropsychological evidence showing that the connections comprising the fornix are required for specific aspects of event memory. The fornix, however, contains many different hippocampal connections, and so our attention then shifts to focus on those fornical connections between the hippocampal formation and the medial diencephalon. The latter region is of particular interest because pathology in this region is closely associated with amnesia, with much evidence linking damage in sites such as the mammillary bodies and the anterior thalamic nuclei to anterograde amnesia.

In the following sections, new research into the anatomical and electrophysiological properties of the hippocampal → anterior thalamic and hippocampal → mammillary body connections will be described, along with the results of novel surgical disconnections within these pathways (an → represents a directional pathway). The overriding goal is to derive a model of how these diencephalic regions interact with the hippocampus and so support learning and memory. Emphasis will be placed on the following emergent concepts: (i) that hippocampal inputs to the medial diencephalon are very precisely arranged to ensure the convergence of parallel, but different, information streams within the anterior thalamic nuclei; (ii) that these medial diencephalic targets are not mere relays for the conveyance of hippocampal information; (iii) that this process of information convergence within the medial diencephalon involves additional afferent pathways, including those from the tegmentum to the mammillary bodies; (iv) that the hippocampal–medial diencephalic interactions supporting memory are reciprocal, i.e. the diencephalon has a vital role in controlling hippocampal function; and (v) that these interactions are vital for episodic memory but are not required for familiarity-based recognition.

## 1. What type of memory is the fornix required for?

### 1.1 Clinical studies

**Fig. 1:**
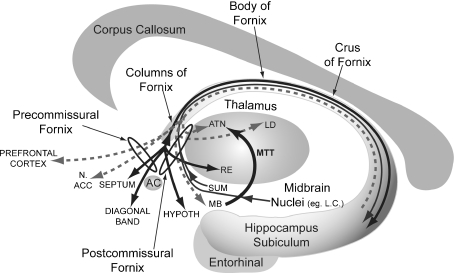
Diagrammatic representation of the location of the fornix and its divisions. The dashed arrows show fornical connections that are solely efferent from the hippocampal formation, the narrow solid arrows show fornical connections that are solely afferent to the hippocampal formation, and the wide solid arrows show reciprocal connections within the fornix. AC, anterior commissure; ATN, anterior thalamic nuclei; HYPOTH, hypothalamus; LC, locus coeruleus; LD, thalamic nucleus lateralis dorsalis; MB, mammillary bodies; MTT, mammillothalamic tract; RE, nucleus reuniens; SUM, supramammillary nucleus.

The fornix emerges from the caudal hippocampus to form an arch in the third ventricle that lies just below the corpus callosum and above the thalamus (Fig. 1). Near its rostral limit the fornix descends at the level of the anterior commissure, where it divides to interconnect the hippocampal formation with an array of subcortical and cortical sites (Fig. 1). Given the size of this tract, e.g., in the human brain the fornix is thought to comprise over 2 000 000 fibres in each hemisphere (Daitz, 1953), and given the numerous sites that it reaches, it is inevitable to ask: (i) whether the fornix is vital for memory; and, if so, (ii) how might the various connections in this tract support memory?

Some years ago there was active debate over whether fornix damage affected human memory at all (for differing views see Garcia-Bengochea & Friedman, 1987; Gaffan & Gaffan, 1991). There is now, however, a clear consensus that fornix damage can permanently impair new learning and memory. This more recent clinical evidence comes from neuropsychological studies that have examined a variety of causes of fornix pathology. Although in none of these studies is the pathology completely restricted to the fornix, it is the one shared feature (Gaffan *et al.*, 1991; D’Esposito *et al.*, 1995; McMackin *et al.*, 1995; Aggleton *et al.*, 2000; Park *et al.*, 2000; Gilboa *et al.*, 2006; Poreh *et al.*, 2006; Vann *et al.*, 2008). While there are some variations in the reported patterns of cognitive change, presumably linked to variations in additional pathology, the core change is a persistent difficulty in learning new episodic information. The task has, therefore, moved on to specifying more precisely the nature of this memory loss and to determine which fornical connections might be the most crucial.

Patients with bilateral fornix damage show consistent memory deficits on both verbal and nonverbal tasks that tax supra-span memory, while short-term memory tasks are spared. A striking feature in a number of reports is the disproportionate loss of episodic memory in contrast to a relative sparing of recognition memory. This pattern was first detected by McMackin *et al.* (1995) in their study of six colloid cyst patients, a condition often associated with fornix damage. A follow-up study with a larger cohort of colloid cyst patients found the same pattern (Aggleton *et al.*, 2000), which has since been seen in cases with other forms of fornix damage (e.g. Vann *et al.*, 2008). One early concern was that recognition memory tasks are typically easier than recall tests, and so potentially prone to ceiling effects, i.e., high scores may be achieved by people who are in reality poor at recognition. Consequently, a person with only a moderate amnesia might appear to have relatively intact recognition. However, this account fails to explain the performance of a man with bilateral fornix damage associated with removal of an angioma (Vann *et al.*, 2008). This person showed moderate, but persistent, deficits on tests of memory recall, but typically appeared to have intact recognition memory. Of particular note was that he displayed completely normal levels of forced-choice recognition memory after a retention delay of 24 h, which was demonstrably sufficient to eliminate ceiling effects (Vann *et al.*, 2008).

Further, relevant evidence comes from a study of 38 people who had all received surgery to remove a colloid cyst in the third ventricle (Tsivilis *et al.*, 2008). Volumetric measures were taken of the fornix, along with an array of other brain sites. The small subset of cases with complete, bilateral severance of the fornix displayed marked episodic memory deficits (Tsivilis *et al.*, 2008). In the remaining colloid cyst cases, the fornix was continuous along the length of the tract, though it often appeared displaced and atrophied. One additional site showing persistent atrophy was the mammillary bodies (Denby *et al.*, 2009). As the mammillary bodies receive dense, direct hippocampal projections via the fornix (Aggleton *et al.*, 2005b), it has been supposed that mammillary body shrinkage partly reflects fornix fibre loss (see Loftus *et al.*, 2000). Correlation analyses using all 38 cases revealed that mammillary body volume consistently predicted performance on tests of recall but not recognition, i.e. small mammillary bodies were selectively and repeatedly associated with poor performance on tests of the recall of episodic information (Tsivilis *et al.*, 2008). The failure to find the same consistent pattern of memory correlations with overall fornix volume may partly stem from the fact that the key measure of fornix atrophy is likely to be its smallest cross-sectional area. While this measure may best quantify the degree of white matter disconnection, it is currently very difficult to calculate fornix cross-sectional area with precision from MRI scans. An additional explanation for the pattern of correlations may prove to be that the mammillary body projections back to the temporal lobe (via the thalamus) are independently important for memory (Vann, 2010), a possibility that is discussed at more length in Sections 4 and 5. Thus, this study (Tsivilis *et al.*, 2008) not only showed a clear dissociation between recall and recognition but also highlighted the potential importance of the mammillary bodies along with the fornix.

**Fig. 2:**
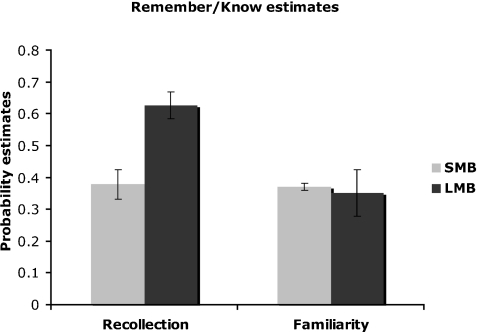
Patients with atrophied mammillary bodies (SMB) show a selective loss of recollective-based recognition, based on the Remember/Know procedure. Derived probability estimates of ‘Recollection’ and ‘Familiarity’ come from a Small Mammillary Body (SMB, *n* = 9) and a Large Mammillary Body (LMB, *n* = 9) group, both drawn from a cohort of post-surgery colloid cyst patients (Vann *et al.*, 2009a,b;). While the two groups differ markedly on ‘Recollection’ (*P* < 0.005), they do not differ on ‘Familiarity’. In a yes/no recognition test participants were asked to make a subjective judgment about their ‘yes’ responses, giving a decision as to whether they were ‘recollective’ in nature (accompanied by other information associated with their initial exposure to the target) or ‘familiar’ in nature (a sense of familiarity devoid of other information). Data presented in histograms are means ± SEM. ‘Familiarity’ is the probability of a familiar response given the item was not recollected (F/(1−R)). Recollection is the probability an old item had a remember response minus the probability that new item had a remember response. To address false alarm rates, familiarity was first estimated for ‘old’ items and for ‘new’ items, and then familiarity for new items was subtracted from familiarity for old items.

The next task is to determine why such patients might show relatively preserved recognition memory. One hypothesis is that the hippocampus and its direct diencephalic connections via the fornix are required for recollection-based recognition but not for familiarity-based recognition (Aggleton & Brown, 1999). This explanation is derived from dual-process models that assume independent mechanisms (and pathways) that can support recognition memory (Mandler, 1980; Yonelinas, 2002). This explanation (a selective loss of recollection-based recognition) received preliminary support from a study of several colloid cyst patients with bilateral fornix loss (Aggleton *et al.*, 2000), though the first direct test in a group study came from the cohort of 38 cases described above (Tsivilis *et al.*, 2008). From this cohort, detailed comparisons (Vann *et al.*, 2009b) were made between the nine patients with the smallest mammillary body volumes and the nine colloid cyst patients with the largest mammillary body volumes (though still less than normal). This comparison ensured that the two groups were very closely matched, apart from mammillary body volume and fornix volume. Convergent findings (Vann *et al.*, 2009b) from three different methodologies (remember/know, receiver operating characteristics, structural equation modelling) were consistent with a loss of recollection-based recognition but a sparing of familiarity-based recognition in those patients with the smaller mammillary body volumes (Fig. 2). Other evidence for this same dissociation comes from single case studies of patients with fornix damage (Gilboa *et al.*, 2006) and mammillothalamic tract damage (Carlesimo *et al.*, 2007), who can also show a disproportionate loss of recollection-based recognition. Additional support for this dissociation comes from an MRI study of normal participants (Rudebeck *et al.*, 2009). In this study, a selective correlation was found between fornix status and memory, as measures of the microstructural integrity of the fornix were associated with recollection-based recognition but not familiarity-based recognition. All of these studies, therefore, support the initial prediction (Aggleton & Brown, 1999) that the fornix is particularly important for recollection-based recognition and that fornix damage spares familiarity-based recognition.

These findings concerning the fornix and its diencephalic projections raise the question of how closely does damage to the fornix mimic the impact of selective hippocampal damage. While some clinical studies find a selective sparing of recognition memory in patients with relatively discrete hippocampal pathology (Yonelinas *et al.*, 2002; Holdstock *et al.*, 2002; Bastin *et al.*, 2004; Aggleton *et al.*, 2005a; Adlam *et al.*, 2009), others report equivalent deficits in recall and recognition (Manns & Squire, 1999; Kopelman *et al.*, 2007; Cipolotti *et al.*, 2006). More detailed analyses have found that those hippocampal pathology cases with a relative sparing of recognition consistently show a disproportionate loss of recollection-based recognition compared to familiarity-based recognition (Holdstock *et al.*, 2002; Bastin *et al.*, 2004; Aggleton *et al.*, 2005a; Gardiner *et al.*, 2006). Those hippocampal cases with consistent recognition memory deficits show a different profile, i.e., deficits for both recollective-based recognition and familiarity-based recognition (Manns *et al.*, 2003; Cipolotti *et al.*, 2006). It has proved very difficult to find a consistent explanation for these discrepancies as there is no obvious factor linked to just those cases with spared recognition, e.g., aetiology of hippocampal pathology, extent of hippocampal atrophy, or age of onset of amnesia. It can, therefore, be seen that at present some patients with relatively circumscribed hippocampal pathology closely resemble the cognitive profile seen after fornix lesions (a selective loss of recall). The problem is that other patients do not fit this pattern. One approach will be to determine whether there are individual patterns of extrahippocampal temporal lobe dysfunction as revealed, for example, by functional MRI that might explain this variability. This same uncertainty underlines the potential value of considering experiments with other animal species.

### 1.2 Animal studies

#### 1.2.1 Recognition memory

The outcome of the clinical findings from patients with fornix damage can be combined with studies into the impact of selective fornix transection in monkeys and rats, where the pathology is more circumscribed and can ultimately be confirmed post-mortem. Studies with monkeys using the delayed nonmatching-to-sample (DNMS) task have often reported that fornix damage has little or no apparent effect on visual recognition memory (e.g. Bachevalier *et al.*, 1985; Zola-Morgan *et al.*, 1989; Charles *et al.*, 2004). Likewise in rats, tests of nonmatching (Shaw & Aggleton, 1993) and spontaneous object recognition (Ennaceur *et al.*, 1997; Bussey *et al.*, 2000b; Clark *et al.*, 2000; Easton *et al.*, 2009) again fail to find deficits after fornix lesions. Such results readily accord with the model of spared familiarity-based recognition in patients with fornix damage if it is assumed that animals are solely or predominantly reliant on familiarity to solve most recognition problems (see Gaffan *et al.*, 1984). Given the patterns of pathology described in the colloid cyst cases above (Tsivilis *et al.*, 2008), it is noteworthy that mammillary body lesions in monkeys (Aggleton & Mishkin, 1985; Zola-Morgan *et al.*, 1989) and in rats (Aggleton *et al.*, 1990, 1995) also largely spare object recognition memory.

Of particular note is a study (Saunders, 1983) that compared the impact of fornix lesions, mammillary body lesions, and fornix plus mammillary body lesions on object recognition (DNMS) performance by cynomolgus monkeys. Once again, both the fornix and mammillary body lesions were associated with only a mild DNMS deficit. Critically, the same result was found for the combined surgery group (fornix plus mammillary bodies), i.e., recognition performance in this group was indistinguishable from the two groups with lesions in just one of these sites. This nonadditive finding is to be predicted if the fornix → mammillary body pathway is not required for familiarity-based recognition (Aggleton & Brown, 2006).

#### 1.2.2 Hippocampal vs. fornix lesions

The next task is to determine whether the fornix is required for all forms of memory that are hippocampal-based. Here, we will principally consider studies with rats as hippocampal-based learning has been so clearly established in this species. Such studies then enable direct comparisons with the impact of fornix damage. Given that the hippocampal connections with cortical sites such as the entorhinal, perirhinal, postrhinal and retrosplenial cortices do not involve the fornix, it might be anticipated that there could be large discrepancies between the impact of hippocampal and fornix lesions. This section will only consider lesion studies involving the entire tract, i.e., surgeries that disconnect both the pre- and postcommissural components of the fornix.

For a wide array of tests of spatial learning, fornix lesions in rats typically echo the outcome of hippocampal lesions, though the deficits can be less severe (Whishaw & Jarrard, 1995; Cassel *et al.*, 1997, 1998; Aggleton & Brown, 2002). An important factor is the specificity of the hippocampal surgery, so that, when hippocampal lesions are more confined to the CA fields and dentate gyrus, the quantitative differences diminish between the effects of hippocampal and fornix lesions (Whishaw & Jarrard, 1995; Whishaw & Tomie, 1997; Aggleton & Brown, 2002). However, it has been suggested that, for some specific classes of learning, the impact of hippocampal damage might differ qualitatively from that of fornix damage. Potential classes of learning showing such a dissociation include recognition memory, spatial conditional learning and configural learning.

As noted above, there is consistent agreement that fornix lesions in rats do not disrupt tests of object recognition memory (e.g. Shaw & Aggleton, 1993; Ennaceur *et al.*, 1997; Bussey *et al.*, 2000b; Clark *et al.*, 2000; Easton *et al.*, 2009). There have been many more studies into the impact of hippocampal lesions in rodents on object recognition, and here the picture is like that reported for clinical studies, i.e. sparing is observed in some, but not all, studies (for reviews see Brown & Aggleton, 2001; Mumby, 2001; Winters *et al.*, 2008). Even so, the conclusion from all of these reviews is that damage to the hippocampus has little, if any, impact on the ability to recognise objects *per se*. The concern is that additional factors, such as changes in exploratory behaviour and the size of the stimuli, might all play a part in producing a hippocampal-related deficit. A related issue is that hippocampal (or fornix) damage in rats may disrupt processes associated with object recognition, such as object-context and object-place learning, thus altering patterns of performance yet sparing familiarity-based discriminations (Ennaceur *et al.*, 1997; Fortin *et al.*, 2004; Winters *et al.*, 2004; Aggleton & Brown, 2006; Easton *et al.*, 2009; Albasser *et al.*, 2010). While these considerations all highlight the complexities of studying recognition memory, there is currently no convincing basis for a dissociation between the impact of hippocampal damage and fornix damage on object recognition itself.

Preliminary evidence suggests that hippocampal, but not fornix, lesions impair spatial conditional learning of the form ‘select stimulus X in place A but select stimulus Y in place B’ (Dumont *et al.*, 2007). Interpreting this difference, however, is complex as studies into the impact of fornix lesions in monkeys have shown that this surgery sometimes impairs and sometimes spares spatial-object conditional learning (Gaffan & Harrison, 1989). The critical difference (Gaffan & Harrison, 1989) appears to be whether the spatial location is specified by the relative spatial arrangement of common items (fornix-sensitive) or whether the location can be identified by unique elements (fornix-insensitive). This distinction relates to configural vs. elemental discrimination problems, and so brings us to the third potential dissociation (configural learning) between the impact of fornix and hippocampal lesions.

**Fig. 3:**
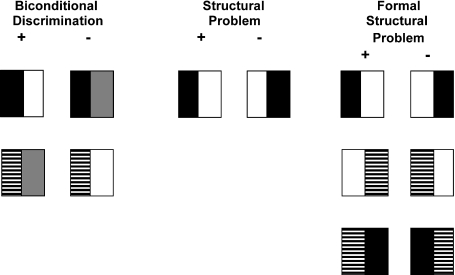
Example stimuli depicting different classes of configural discrimination. Left: biconditional discrimination, where all four elements A, B, X, Y, are found equally in the rewarded (+) and nonrewarded (−) compound stimuli. Middle: depiction of the structural discrimination, A to the left of X vs. X to the left of A. Note, this task can be solved nonstructurally if the subject always attends to just the extreme left (or right) element of the compound stimulus as now it can be solved elementally using A− and X+. Right: formal set of concurrent structural discriminations that are used to ensure that subjects cannot solve the task merely by attending to just one side of each compound stimulus.

In configural learning, the subject cannot solve the discrimination task by reference to a single element. Instead, the subject must learn the specific pairings of elements to solve the problem. An example of a configural discrimination is the biconditional problem, e.g. AX+ BY+ AY− BX− (+ signifies reward and − signifies no reward). It can be seen (Fig. 3, left) that no single element (A, black; B, horizontal lines; X, white; Y, grey) is consistently associated with reward and that the specific pairings (configurations) must be learnt. Fornix lesions in rats typically have no impact on tests of configural learning (Whishaw & Tomie, 1995; McDonald *et al.*, 1997; Bussey *et al.*, 1998, 2000a), yet the same classes of test can be impaired by hippocampal lesions (e.g., Sutherland & Rudy, 1989; McDonald *et al.*, 1997; Driscoll *et al.*, 2005; Iordanova *et al.*, 2009). Remarkably, in some studies, fornix lesions have been associated with enhanced configural learning (Bussey *et al.*, 1998; Aggleton *et al.*, 2009), so further underlining this potential dissociation. One possible explanation for this enhancement is that this surgery biases processing towards the parahippocampal region and away from the hippocampus, with the former region being of particular importance for stimulus–stimulus configurations (Bussey *et al.*, 2002). There is, however, an important limitation with this possible dissociation between fornix and hippocampus over configural learning: hippocampal lesions do not always impair such learning (e.g., Gallagher & Holland, 1992; Davidson *et al.*, 1993; Sanderson *et al.*, 2006). As a consequence, there is a need to define more precisely the properties of those configural tasks that are consistently disrupted by hippocampal damage.

One proposal is that hippocampal lesions consistently impair a specific class of configural task that has been called ‘structural’ (Aggleton *et al.*, 2007). The term ‘structural’ refers to the ability to learn the precise spatial (or temporal) relationships between the various elements that comprise the overall complex array, i.e. the structure of each element with reference to its surroundings. The prototypical example would be a discrimination of the form black to the left of white vs. black to the right of white (Fig. 3, middle), as this configural discrimination additionally requires the embedded dimension of spatial information. This discrimination can be described more generally as A to the left of X (A|X) vs. A to the right of X (X|A). Testing this ability unambiguously requires a more complex set of concurrent discriminations in order to preclude nonconfigural strategies (Fig. 3, right). Like all configural tasks, it can be seen (Fig. 3, right) that no single element (black, white or horizontal lines) can solve the task, and while particular pairings of elements (A with X, X with Y, Y with A) must be learnt, this is still insufficient. Thus, while most configural tasks work at this latter level (specific combinations between A, X, Y, etc.), structural discriminations have an additional spatial or temporal component, e.g., horizontal to the left of black vs. horizontal to the right of black.

While rats with hippocampal damage are impaired at performing such ‘structural’ tasks, they can acquire at a normal rate other configural tasks using the same apparatus and the same visual elements, e.g., biconditional learning (Sanderson *et al.*, 2006). In contrast to the hippocampal deficit, fornix damage was found to facilitate learning the same structural discrimination (Aggleton *et al.*, 2009). Intriguingly, a similar enhancement after fornix damage had previously been reported for a task that also required object–place conjunction learning (Gaffan *et al.*, 2001), a feature that lies at the heart of structural learning. The implication is that structural learning is reliant on corticohippocampal interactions that do not require the fornix.

A qualification emerging out of these structural discriminations is the contrast between one-trial learning and incremental learning over many sessions. Those tests that formally tax structural learning by rats and are spared by lesions of the fornix (Gaffan *et al.*, 2001; Aggleton *et al.*, 2009) require multiple training trials. These results can be contrasted with less rigorous one-trial tests that examine learning the relationship between a specific object and its spatial or temporal location. Here, the situation seems different, as studies with rats (object-in-position, item recency) can show fornix lesion-induced impairments (Ennaceur *et al.*, 1997; Bussey *et al.*, 2000b). Likewise, studies with monkeys reveal deficits for ‘object-in-place’ learning after fornix lesions (Parker & Gaffan, 1997b) using a task that involves rapid discrimination learning. The identical task has been given to patients with fornix damage (Aggleton *et al.*, 2000), and the profile of object-in-place deficits very closely matches that observed in monkeys with fornix lesions, suggesting a close link between the loss of episodic memory in humans and such learning in animals.

In summary, both human and animal studies show that the connections that comprise the fornix are vital for memory, but that they are only required for specific forms of memory. While familiarity-based recognition appears to be preserved after fornix damage in humans, clear deficits are found in tests requiring the recall of episodic information. These recollection deficits correlate closely with the status of the mammillary bodies. However, it should be noted that impairments on tests of recollection-based recognition do not necessarily mean that the principal deficit is one of retrieval *per se*. For example, dual-process models that assume independent familiarity and recollective based mechanisms predict that the poor encoding of information for the latter process will also disrupt recall.

The apparent sparing of recognition memory after fornix damage is also found in animal studies, though here it is more difficult to separate familiarity from recollection-like processes (Fortin *et al.*, 2004; Easton *et al.*, 2009). Even so, ingenious attempts to separate these processes again indicate that both fornix (Easton *et al.*, 2009) and hippocampal (Fortin *et al.*, 2004) lesions in rats leave familiarity intact but impair recollection-like processes. It can also be shown that the fornix is important for spatial learning, though the loss of this tract does not completely block place learning (Whishaw & Jarrard, 1995; Whishaw & Tomie, 1997). As noted above, animal studies offer the chance to compare directly the impact of hippocampal lesions with fornix lesions, and so determine whether there are forms of hippocampal-based learning that do not require the connections in the fornix. While three different classes of learning were considered (recognition, spatial conditional learning and configural learning), in no single case is there yet sufficient evidence to conclude that the effects of fornix and hippocampal lesions differ qualitatively though this must remain a possibility. One possible exception is gradual (fornix-insensitive, hippocampal-sensitive) vs. rapid (both fornix- and hippocampal-sensitive) learning of object–location configurations. When making such comparisons, great care has to be taken over defining the extent of the hippocampal lesion, not only to rule out the contributions from any extrahippocampal damage but also to consider whether the subiculum is included in the hippocampal lesion as this may potentiate the impact of a more selective hippocampal lesion (Morris *et al.*, 1990). The significance of the latter factor arises from the fact that the subiculum has its own connections that are independent of the hippocampus proper, in addition to being the link for very many hippocampal connections, including those with the medial diencephalon.

## 2. Hippocampal projections to the medial diencephalon

In order to appreciate why the fornix is important for some forms of learning, it is necessary to consider in more detail the connections that use this tract (Fig. 1). As already noted, the hippocampal formation is directly linked via the fornix with numerous brain sites. For some sites, the fornix contains only efferent hippocampal fibres, e.g., to the anterior thalamic nuclei, mammillary bodies, ventral striatum and prefrontal cortex (Saunders & Aggleton, 2007). For other targets, the hippocampal connections within the fornix are reciprocal, e.g. with the medial septum and nucleus reuniens (Fig. 1). Last, for some sites, the fornix appears to contain fibres that are solely afferent to the hippocampus, e.g., from the supramammillary nucleus, the raphe nucleus and the locus coeruleus (Swanson *et al.*, 1987; Saunders & Aggleton, 2007). The next task is to distinguish the functional contributions of these various fornical connections. Although we will seek to test the contributions of two particular sets of hippocampal connections, this exercise is not exclusive, i.e. demonstrating the importance of one set of connections does not rule out the importance of other connections.

In this review, our focus will be on the hippocampal connections with two sites, the anterior thalamic nuclei (defined as comprising the anterior medial, anterior ventral and anterior dorsal thalamic nuclei) and the mammillary bodies, as there is overwhelming evidence to suggest that these particular connections are critical for memory. This conclusion principally follows from a consideration of the pathology of anterograde amnesia (Aggleton & Saunders, 1997). While it is accepted that bilateral hippocampal damage can cause temporal lobe amnesia (Spiers *et al.*, 2001), diencephalic amnesia is most consistently associated with pathology in the mammillary bodies, the mammillothalamic tract and the anterior thalamic nuclei (Von Cramon *et al.*, 1985; Dusoir *et al.*, 1990; Clarke *et al.*, 1994; Ghika-Schmid & Bogousslavsky, 2000; Harding *et al.*, 2000; Van der Werf *et al.*, 2000, 2003; Gold & Squire, 2006). The focus on these particular diencephalic structures does not mean that other diencephalic sites do not contribute to this amnesic syndrome, but rather that damage in these target regions is most likely to be responsible for the core memory deficits observed.

The underlying assumption of an important functional linkage between the hippocampus and these medial diencephalic sites is strongly supported by studies with rats showing how lesions in the hippocampus, fornix, mammillary bodies and anterior thalamic nuclei all disrupt the same tests of spatial learning, though with different degrees of severity (Aggleton & Sahgal, 1993; Aggleton *et al.*, 1995; Byatt *et al.,* 1996; Sziklas & Petrides, 1998; Vann & Aggleton, 2003). In monkeys, lesion studies again strongly suggest that the fornix, mammillary bodies and anterior thalamic nuclei function together in the learning of visual discriminations (‘object-in-place’) that are aided by contextual information (Parker & Gaffan, 1997a,b;). A further similarity between the impact of lesions in the hippocampus, fornix and anterior thalamus in animals is that they can all disrupt tests of temporal order discrimination (Fortin *et al.*, 2002; Charles *et al.*, 2004; Wolff *et al.*, 2006). Finally, crossed-lesion disconnection studies add further weight to the notion that the hippocampus and anterior thalamic nuclei depend on each other for effective spatial learning (Warburton *et al.*, 2000, 2001; Henry *et al.*, 2004).

From Fig. 1, it can be seen that the hippocampal formation projects directly to both the anterior thalamic nuclei and the mammillary bodies. A key discovery was that the hippocampal projections to the medial diencephalon arise not from the hippocampus proper but from the subiculum (Figs 1 and 4; see also Swanson & Cowan, 1975; Rosene & Van Hoesen, 1977; Aggleton *et al.*, 1986). In the monkey, all of the hippocampal projections to the mammillary bodies require the fornix (Aggleton *et al.*, 2005b), and the same is true of the overwhelming majority of the hippocampal projections to the anterior thalamic nuclei (Aggleton *et al.*, 1986; Saunders *et al.*, 2005).

**Fig. 5:**
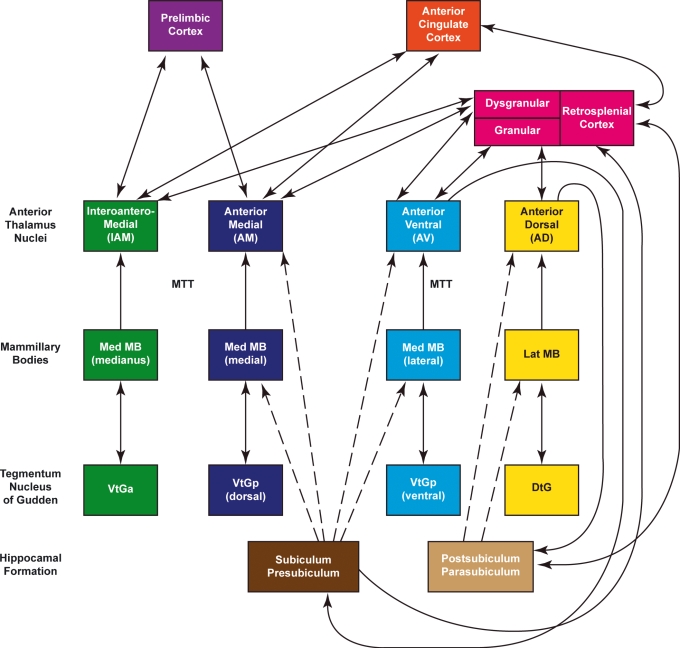
Diagram of interlinked connections in the rat brain showing how the hippocampal formation is associated with three distinct sets of parallel anterior thalamic connections. Connections conveyed in the fornix are shown as dashed lines. Double-headed arrows depict reciprocal connections. The connectivity of the interoanteromedial nucleus is taken from Hopkins (2005). DtG, dorsal tegmental nucleus of Gudden; MTT, mammillothalamic tract; VtGa, ventral tegmental nucleus of Gudden, pars anterior; VtGp, ventral tegmental nucleus of Gudden, pars posterior.

It is also evident from Fig. 1 that the hippocampus projects both directly and indirectly to the anterior thalamic nuclei as the mammillary bodies innervate the anterior thalamic nucleus via the mammillothalamic tract. The large scale of the mammillothalamic tract projections is underlined by evidence that almost every mammillary body cell projects to the anterior thalamus (Hopkins, 2005; Vann *et al.*, 2007) and by the absence of interneurons in this structure. Other major mammillary efferent projections, e.g., to the tegmentum, arise from bifurcating neurons that project to both the thalamus and the tegmentum (Takeuchi *et al.*, 1985). The mammillothalamic projections show an intriguing degree of organisation in the primate brain as all parts of the medial mammillary nucleus project ipsilaterally to the region of the anterior medial thalamic nucleus, while the projections to the anterior ventral nucleus arise predominantly from a distinct, dorsal cap to the medial mammillary nucleus (Vann *et al.*, 2007). In the rat brain, these topographies have been studied in finer detail (Fig. 5), revealing that inputs to the anterior medial and anterior ventral thalamic nuclei arise from different parts of the medial mammillary nucleus, though there is some minor disagreement over the precise arrangements (Shibata, 1992; Hopkins, 2005). It is, however, agreed that the lateral mammillary nucleus in both the monkey and rat brain is the sole source of the mammillary projections to the anterior dorsal thalamic nucleus (Shibata, 1992; Hopkins, 2005; Vann *et al.*, 2007), with individual lateral mammillary neurons bifurcating to project both ipsilaterally and contralaterally to the anterior dorsal nucleus (Kuypers *et al.*, 1980). One implication is that in the monkey, as in the rat, the three anterior thalamic nuclei are likely to have different functional roles given their contrasting patterns of mammillary body innervation (Hopkins, 2005; Vann *et al.*, 2007).

**Fig. 4:**
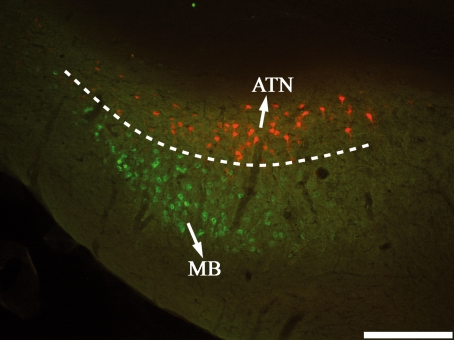
Contrasting distribution of dorsal subicular cells projecting to the anterior thalamic nuclei (ATN) and mammillary bodies (MB). Coronal colour section through the dorsal subiculum (right hemisphere) showing the separate populations of subicular cells projecting to the anterior thalamic nuclei (red cells, fast-blue injection) and the mammillary bodies (green cells, cholera toxin-488 injection). The dashed line shows the division, with those cells closest to the alveus (upper) projecting to the thalamus (ATN). White scale bar, 250 μm.

In order to model how the hippocampus, mammillary bodies and anterior thalamic nuclei might function in concert, it is necessary to consider in much finer detail the nature of their respective connections. As noted above, a crucial discovery was that the hippocampal projections to the medial diencephalon originate in the subicular cortices. Studies in both the rat and monkey suggest that separate populations of cells within the subiculum give rise to the respective projections to the mammillary bodies and anterior thalamus (Naber & Witter, 1998; Ishizuka, 2001; Aggleton *et al.*, 2005b). To test this suggestion formally, we placed different fluorescent retrograde tracers in the anterior thalamic nuclei and mammillary bodies of the same rats (Wright *et al.*, 2010). The results were striking, revealing two segregated bands of limbic projections, one to the anterior thalamic nuclei and one to the mammillary bodies (Fig. 4).

The anterior thalamic nuclei were found to receive numerous projections from a continuous dorsoventral band of limbic cortex involving the retrosplenial cortex, subiculum and postsubiculum (Wright *et al.*, 2010). Additional anterior thalamic inputs arose from the entorhinal cortex, though these projections were far less frequent. [Note, all of these projections, except those from the retrosplenial cortex (Meibach & Siegel, 1977), rely on the fornix as a route]. Immediately adjacent to these limbic cells that project to the thalamus, numerous other cells project to the mammillary bodies from the subiculum, postsubiculum and entorhinal cortex (Fig. 4). Apart from a very limited number of individual cells that project to both the mammillary bodies and the anterior medial thalamic nucleus, these two dense bands of limbic cells remain completely separate. The thalamic projections arise from subicular cells that lie deep to those that project to the mammillary bodies (Wright *et al.*, 2010). While only pyramidal cells appear to project to the mammillary bodies, deeper pyramidal and polymorphic cells project to the anterior thalamic nuclei. Other anatomical studies suggest that, for the subiculum, this segregated organisation applies to a much wider array of efferents, i.e. that subicular outputs are typically parallel in origin (Naber & Witter, 1998; Ishizuka, 2001). In contrast, efferents from the CA1 field appear to show an appreciably higher degree of collateralization (Naber & Witter, 1998).

An emerging feature of the limbic projections to the medial diencephalon is, therefore, the way in which they can potentially provide convergence from parallel, but different, information streams. While separate cell populations in the hippocampal formation innervate the anterior thalamic nuclei and the mammillary bodies (Wright *et al.*, 2010), it has been noted that, within the rat brain, the hippocampal projections to the mammillary bodies and those from the tegmental nuclei of Gudden to the mammillary bodies have orthogonal patterns of topography, so ensuring the fullest overlapping of these two afferent sources (Hopkins, 2005). At this point, it is helpful to distinguish the lateral mammillary body from the medial mammillary body systems (Vann & Aggleton, 2004). The lateral mammillary nucleus is innervated by the dorsal tegmental nucleus of Gudden and projects to the anterior dorsal thalamic nucleus. This circuitry is crucial for ‘head-direction’ information. The medial mammillary nucleus, however, is innervated by the ventral tegmental nucleus of Gudden and projects to the anterior medial and anterior ventral thalamic nuclei. As depicted in Fig. 5, this medial system can be further subdivided in the rat brain, each subdivision with its own distinct patterns of connectivity. The two major systems (medial and lateral), and their subsystems (Fig. 5), appear to remain separate within the diencephalon, with the subsequent potential for their convergence in sites such as the retrosplenial and subicular cortices.

In the rat, an additional subsystem within the medial mammillary body system has been identified (Fig. 5). This subsystem centres on pars medianus of the medial mammillary nucleus (Hopkins, 2005). Pars medianus has its own tegmental inputs (from pars anterior of the ventral tegmental nucleus of Gudden) and its own thalamic projections (to the interanteromedial thalamic nucleus). Pars medianus may, however, lack direct hippocampal formation inputs (Hopkins, 2005), so giving it quite separate properties. It is also uncertain whether an analogous system exists in the primate brain as anatomists have often failed to distinguish a pars medianus for the mammillary bodies or a distinct interanteromedial thalamic nucleus in the monkey brain. A more likely additional system in the primate brain involves the lateral dorsal thalamic nucleus. This nucleus is sometimes regarded as an extra ‘anterior thalamic’ nucleus as it also has dense interconnections with the hippocampal formation and cingulate cortices. The lateral dorsal thalamic nucleus, however, receives very few inputs from the mammillary bodies (Vann *et al.*, 2007), and for this reason will not be considered further in this review.

An outstanding, but unresolved, question is whether the subiculum is organised with truly independent outputs. While our recent anatomical studies highlight the scale of the parallel limbic inputs to the mammillary bodies and anterior thalamus (Wright *et al.*, 2010), it is not known whether the two distinct populations of subicular cells also receive independent afferent information or whether they share common sources of information (Naber & Witter, 1998). Evidence that many of the subicular cells projecting to the mammillary bodies bifurcate to project also to the entorhinal cortex, a feature not found in those subicular cells projecting to the thalamus (Donovan & Wyss, 1983), suggests that these separate cell populations may prove to be independent, as they have different properties. Other evidence comes from *in vitro* recording studies showing that different depths of cells within the ventral subiculum have both distinct morphological and electrophysiological properties (Greene & Totterdell, 1997). Further evidence of qualitative differences between the hippocampal → anterior thalamic connections and the mammillary body→ anterior thalamic connections comes from the *in vivo* electrophysiological studies that are described in the next section. At the same time, it must be remembered that the mammillary bodies project very densely across the anterior thalamic nuclei, i.e., the separate hippocampal → anterior thalamic nuclei and hippocampal → mammillary body projections potentially converge in the anterior thalamus after just one relay in the latter (hippocampal → mammillary body) pathway.

## 3. The contrasting electrophysiological properties of the hippocampal → anterior thalamic pathway vs. the mammillary body→ anterior thalamic pathway

Synaptic plasticity is a ubiquitous property of central synapses which has been widely thought to have properties consistent with the requirements of a neurobiological information storage mechanism (Bliss & Collingridge, 1993; Barnes, 1995; Bear, 2003; Morris, 2003). For synaptic plasticity to be an information storage mechanism, plastic changes must persist long enough to form the basis for long-term memory instantiation (minimally tens of minutes, to hours or more). Two key forms of synaptic plasticity, long-term potentiation (LTP; the activity-dependent enhancement of synaptic strength) and long-term depression (LTD; the activity-dependent decrement of synaptic strength) have been widely investigated in relation to memory function, particularly in the hippocampus itself. LTP is commonly induced by high-frequency stimulation, whereas LTD is generally induced via low-frequency stimulation. A key hypothesis underpinning the idea that synaptic plasticity plays a central role in the biology of information storage is that activity-dependent change is both necessary and sufficient to support memory (e.g., Morris, 2003). We have argued here that the extended hippocampal–diencephalic system, embracing the anterior thalamic nuclei, the medial mammillary bodies and other closely connected structures, is vital for memory, and for long-term memory in particular. Here, we discuss the few data available bearing on the question of activity-dependent change in the extended hippocampal–diencephalic system itself, and we summarise some of our recently-reported data on activity-dependent synaptic plasticity in this system.

Thalamic neurophysiological responsivity is commonly regarded as a gating mechanism regulating corticopetal information flow; this responsiveness is controlled in a state-dependent manner and is dependent on the prior history of thalamic activity (Steriade & Llinas, 1988). Extracellular recordings in urethane-anesthetized cats have already revealed that brain-stem stimulation can induce short-term changes in synaptic plasticity in the anterior thalamic nuclei, i.e. changes lasting minutes (Pare *et al.*, 1990). Simulation of the laterodorsal tegmental nucleus was found to evoke short-latency (10–20 ms) excitation in the majority of recorded anterior thalamic cells (Pare *et al.*, 1990). Critically, brief tegmental stimulation trains enhanced the responsiveness of anterior thalamic cells to both mammillary (orthodromic) and cortical (ortho- and antidromic) stimuli, with these increases in synaptic responsiveness reaching a peak 40–50 s after the stimulation trains and lasting up to 4 min (Pare *et al.*, 1990).

We have more recently investigated the effects of high- and low-frequency stimulation of either the fornix or the mammillothalamic tract in urethane-anaesthetized rats and found that synapses in the anterior thalamic nuclei can undergo both LTP and LTD (of at least 4 h duration; Tsanov *et al.*, 2010). A comparison of the synaptic changes evoked via the direct (fornical) vs. the indirect (via the mammillary bodies) hippocampal pathways to the anterior thalamic nuclei demonstrates that LTP of the thalamic field response is induced predominantly through the mammillothalamic tract (Tsanov *et al.*, 2010). Furthermore, we have found that LTD can only be induced by stimulation of the direct hippocampal (fornical) projections (see Table 1). The complementary properties of these two parallel pathways on anterior thalamic activity strongly suggest that they do not have duplicate functions. This view is supported by the differences in short-term plasticity of the thalamic field potential in response to mammillothalamic tract low-frequency stimulation vs. that induced by direct fornix stimulation. Pulses delivered with a frequency of 1 Hz induced immediate frequency suppression of the field potential slope and amplitude of the fornical output to the anterior thalamus (Tsanov *et al.*, 2010). The same low-frequency stimulation did not evoke frequency suppression of mammillothalamic synapses (Table 1). We conclude, therefore, that the anterior thalamus does not passively relay incoming information, but rather acts as a synaptic network, where the ability to integrate hippocampal and mammillary body inputs is dynamically modified as a result of previous activity in the circuit.

**Table 1:** Comparison of synaptic plasticity in the direct and the indirect hippocampal afferents to anterior thalamus

		Response to			
Stimulated pathway	BDNF dependence	Paired-pulse stimulation	1-Hz stimulation	HFS	LFS
Dfx	No	Facilitation	Suppression of the FP slope and amplitude	LTP of the FP slope No effect on the FP amplitude	LTD of the FP slope and amplitude
MTT	Yes	Facilitation	Suppression of the FP slope No effect on the FP amplitude	LTP of the FP slope and amplitude	LTP of the FP slope and amplitude

The opposing synaptic properties of the hippocampothalamic (dorsal fornix; Dfx) and mammillothalamic tract (MTT) pathways are represented in their basal synaptic transmission, as well as short- and long-term plasticity modifications. A use-dependent baseline augmentation is observed only at mammillothalamic synapses. Paired-pulse facilitation was present for 20-, 30- and 40-ms interstimulus intervals for the field potential (FP) amplitude of the MTT-evoked responses; Dfx paired pulses induced facilitation for 20- and 30-ms intervals. 1-Hz frequency stimulation of MTT stimulation did not affect FP amplitude, while Dfx stimuli induced a gradually developing decrease in the anterior thalamic nuclei (ATN) response. FP amplitude of the thalamic response underwent long-term potentiation (LTP) after high-frequency stimulation (HFS) application to MTT, but not to Dfx. Low-frequency stimulation (LFS) of Dfx induced stable long-term depression (LTD) of the FP parameters; however, the same stimulation protocol applied to MTT evoked mild LTP (Tsanov *et al*., 2010).

In addition to LTP and LTD, we have also examined possible plasticity properties of basal synaptic transmission in the direct and indirect hippocampal inputs to the anterior thalamus (Table 1). Interestingly, we found that basal synaptic transmission mediated by the mammillothalamic tract (but not the fornix) undergoes activation-dependent, brain-derived neurotrophic factor (BDNF)-mediated potentiation. This potentiation appears to be a distinct form of plasticity specific to the diencephalic region (Tsanov *et al.*, 2010). A similar type of thalamic plasticity has already been observed for sensory thalamocortical synapses, where synaptic transmission undergoes potentiation mediated by tyrosine receptor kinase B receptors (which bind BDNF; Tsanov & Manahan-Vaughan, 2007). Cumulatively, these data suggest that input-specific synaptic plasticity is one of the major properties underlying thalamic function in the adult brain (Rauschecker, 1998).

A characteristic feature of anterior thalamic neurons, compared to the rest of the thalamus, is their ability to fire rhythmically in the 5–12 Hz range (the frequency known as theta rhythm; Buzsáki, 2002). Single-unit recordings from the anterior thalamic nuclei in urethane-anaesthetized rats indicate that anterior ventral neurons tend to fire in a fashion highly correlated with theta rhythm (Vertes *et al.*, 2001). A much smaller subset of cells in the anterior medial and anterior dorsal thalamic nuclei also possess rhythmical discharge in the theta range (Albo *et al.*, 2003). These theta oscillations in the anterior ventral nucleus of the thalamus are placed so that they receive descending inputs from the subiculum and ascending inputs from the medial mammillary bodies, thus comprising central components of the medial hippocampal–diencephalic system (see Fig. 5; Vann & Aggleton, 2004). Theta rhythm, which shows increases in power during behavioural arousal, locomotion and REM sleep, is thought to serve a critical role in the mnemonic functions of the limbic system (Vertes & Kocsis, 1997; Kahana *et al.*, 2001; Burgess *et al.*, 2002; Hasselmo *et al.*, 2002; Kirk & Mackay, 2003). It has been proposed that oscillatory patterns in the theta range enable synaptic plasticity (Mehta *et al.*, 2002). Consistent with this view, electrophysiological studies in rats have found that plasticity occurs between sequentially-activated hippocampal place cells during theta epochs (Skaggs *et al.*, 1996; Mehta *et al.*, 2000; Ekstrom *et al.*, 2001; Mehta *et al.*, 2002), thus implicating the theta cycle as an information quantum (Buzsáki, 2002). Furthermore, inactivation of the medial septum abolishes theta rhythmical discharge in both the hippocampus and mammillary bodies (Bland *et al.,* 1995; Kirk *et al.,* 1996), two of the major regions providing inputs to anterior thalamus. Hence, the anterior thalamic nuclei appear to be part of a descending system driven from the medial septum, and theta oscillations in the anterior thalamus might complement hippocampal–diencephalic memory processing (Vann & Aggleton, 2004; Vertes *et al.*, 2004). Future research with freely-behaving animals should verify this hypothesis. At the same time, another potentially important contributor to this theta system is the ventral tegmental nucleus of Gudden (Vann, 2009), with evidence for the importance of this nucleus coming from both targeted lesions and selective tract disconnections within the medial diencephalon (see Section 4).

In summary, there are significant differences in basal synaptic transmission, and short- and long-term synaptic plasticity, in the hippocampal–thalamic and mammillothalamic tracts. A BDNF-dependent augmentation of synaptic transmission is observed only at mammillothalamic synapses. Paired-pulse stimulation, however, induced facilitation in both pathways. Short-term plasticity, induced by 1-Hz frequency stimulation, resulted in suppression of the thalamic field potential amplitude when applied to the dorsal fornix but not to the mammillothalamic tract. In contrast, the amplitude of the thalamic activity is readily potentiated after high-frequency stimulation of the mammillothalamic tract but not of the dorsal fornix. Low-frequency stimulation of dorsal fornix induced stable LTD of the field potential parameters, whereas the same stimulation protocol of the mammillothalamic tract induced potentiation (Tsanov *et al.*, 2010). The overwhelming picture is one in which the two major inputs to the anterior thalamic nuclei have opposing or complementary actions, but they do not duplicate one another.

## 4. The behavioural impact of selective disconnections within the medial temporal–medial diencephalic system

The system under consideration contains discrete fibre tracts linking the nuclei of interest. As a consequence, it is possible to disconnect particular pairs of sites and measure the impact on behavioural tests of learning. So far, we have just considered the behavioural effects of complete fornix lesions. In this section, we will consider disconnections of: (i) just that part of the fornix that projects to the mammillary bodies (lesions of the descending postcommissural fornix), and (ii) the projections from the mammillary bodies to the anterior thalamus (lesions of the mammillothalamic tract). We can predict that both surgeries will produce clear and equivalent deficits on tests of spatial memory if the critical mnemonic system is the serial route from the hippocampus to the mammillary bodies and, thence, to the anterior thalamus (Delay & Brion, 1969; Aggleton & Brown, 1999).

Testing the impact of tract disconnections is not the same as comparing the effects of removing the various sites within the putative system. The difference arises because tract section need not lead to cell death within the sites linked by the tract, so that a target structure may still function effectively due to other preserved inputs. For example, in the systems under investigation, fornix damage does not produce retrograde degeneration in the hippocampus nor does it produce cell loss in the mammillary bodies, despite shrinkage due to the loss of incoming fibres (Loftus *et al.*, 2000).

As the columns of the fornix descend in front of the thalamus to enter the septum (Fig. 1) the pathway divides, with some fibres passing in front of the anterior commissure (precommissural) and others passing caudal to the anterior commissure (postcommissural). As a consequence it would be desirable to compare the impact of selective precommissural vs. postcommissural fornix lesions on learning and memory (Henderson & Greene, 1977; Thomas, 1978). This task has, in fact, proved very difficult as such lesions need to be made at the level of the septum (where the pathways split) yet damage to this site will involve much of the septum itself. An opportunity is, however, provided by the fact that the postcommissural pathway divides again, with many fibres turning immediately caudal to stream directly into the frontal pole of the anterior thalamus while a separate tract descends through the hypothalamus to reach the mammillary bodies (Fig. 1). Estimates of the numbers of fibres in this descending fornical component that leads to the mammillary bodies suggest that, in the human brain, this pathway may contain over one third of all of the fibres in the subcallosal fornix (Daitz, 1953). By selectively sectioning this latter descending pathway, it is possible to disconnect the subicular projections to the mammillary bodies and yet spare the hippocampal connections with sites such as the septum and anterior thalamic nuclei.

In view of the large scale of the hippocampal projections to the mammillary bodies and the importance of both regions for spatial learning, it might be predicted that cutting the descending postcommissural fornix would: (i) consistently disrupt spatial learning, and (ii) produce an impairment of similar extent to that seen after mammillary body (or mammillothalamic tract) damage. Surprisingly, neither of these predictions appears correct (Vann *et al.*, 2010). Despite completely disconnecting the hippocampal → mammillary body projections, the impact of this surgery on spatial learning proved to be remarkably slight, as only mild deficits were apparent on T-maze alternation, while acquisition of spatial working memory tasks in the radial-arm maze and the Morris water maze were not impaired (Vann *et al.*, 2010). These effects appear to be appreciably less severe than those seen after mammillary body lesions (Vann & Aggleton, 2003).

One obvious explanation for the very minor effects seen after hippocampal → mammillary body disconnections is that spatial performance is normally supported by the hippocampal formation inputs to both the anterior thalamus and the mammillary bodies, such that the direct hippocampal → anterior thalamic connections can compensate for the loss of the indirect hippocampal → mammillary → anterior thalamic nuclei route. Two pieces of evidence, however, argue against this explanation. The first is that lesions of the mammillary bodies and of the mammillothalamic tract produce spatial learning deficits that are equivalent to each other, but are more severe than any of those seen after descending postcommissural fornix lesions (Vann & Aggleton, 2003; Vann *et al.*, 2010). This pattern of results is completely at odds with the explanation that the direct hippocampal → anterior thalamic projections are effective at compensating for the loss of the hippocampal → mammillary body projections as in all three surgeries (mammillary body, mammillothalamic tract, descending postcommissural fornix), this same direct hippocampal → anterior thalamic compensation should occur. The second piece of evidence is that the electrophysiological properties of these two sets of hippocampal inputs (direct and indirect) within the anterior thalamic nuclei are strikingly different (see Table 1), making it difficult to envisage a natural compensation process (Tsanov *et al.*, 2010). The surprising conclusions are that: (i) despite the strength of the direct hippocampal projections to the mammillary bodies, the mammillary bodies can function effectively in their absence, and (ii) to disrupt anterior thalamic function the nuclei must lose their direct mammillary body (mammillothalamic tract) inputs rather than their indirect hippocampal inputs via the mammillary bodies. These conclusions also point to the importance of the integrity of mammillary body neurons, especially as the surgical procedure with the smallest behavioural effect (descending postcommissural fornix lesion) does not appear to cause cell loss and atrophy within the mammillary bodies (Loftus *et al.*, 2000; Vann, 2009). In contrast, mammillothalamic tract section produces widespread retrograde degeneration within the mammillary bodies.

The above pattern of results shows that the mammillary bodies cannot simply be considered as a relay for hippocampal information, thus revealing the functional importance of other inputs to the mammillary bodies. Among these inputs are signals to the lateral mammillary nucleus from the dorsal tegmental nuclei of Gudden that comprise part of the ‘head-direction’ system (Hopkins, 2005; Vann & Aggleton, 2004). The firing of neurons in this system provides compass-like information, i.e. the firing is selective for head direction but is not tied to a particular location (Taube, 1998). Therefore, it is natural to assume that such head-direction information contributes to navigation, so accounting for the independent importance of the mammillary bodies, i.e. in the absence of their hippocampal inputs. Current findings on the impact of selective lateral mammillary body lesions only partially support this conclusion (Vann, 2005) as these selective lesions do not mimic the impact of complete mammillary body damage (Vann & Aggleton, 2003). It is, therefore, most likely that the medial mammillary nucleus also has an important role for memory, which again could be partially independent of the hippocampus (Vann, 2010). Indeed, neuropathological studies of the amnesic Korsakoff’s syndrome (Harding *et al.*, 2000; Victor *et al.*, 1971) more strongly implicate the medial mammillary nucleus than the lateral mammillary nucleus, along with those anterior thalamic nuclei (anterior medial and anterior ventral thalamic) that are innervated by the medial mammillary nucleus. If it is the case that the rodent medial mammillary nucleus can support spatial memory in the absence of its hippocampal inputs, we need to identify those other (nonhippocampal) brain sites that enable mammillary body function (Vann, 2010).

One site reciprocally interlinked with the medial mammillary bodies is the ventral tegmental nucleus of Gudden (Hopkins, 2005). This connectivity raises the possibility that this nucleus may be a vital source of extrinsic inputs that can support spatial processing within the medial mammillary bodies. Until recently, nothing was known about the impact of selective lesions of the ventral tegmental nucleus of Gudden, but it is now evident that such lesions can induce very clear deficits on an array of spatial learning tasks that are also sensitive to mammillary body and anterior thalamic damage (Vann, 2009). These deficits imply that the ventral tegmental nucleus may have a largely overlooked role in maintaining mammillary body function, allowing it to support spatial memory in the absence of hippocampal inputs (Vann, 2009). Further testing of this particular idea is clearly required as the ventral tegmental nucleus of Gudden projects to other sites that could potentially aid memory. This revised view also requires us to consider whether the extended hippocampal system is reciprocal, i.e., how the medial diencephalon might act back upon the medial temporal lobe. This issue is considered in Section 5.

## 5. Reversing the picture: measuring the impact of the medial diencephalon upon the hippocampal formation

There is an emerging view, advocated by Vann (2009, 2010), that it is equally, or possibly more, important to consider how the anterior thalamic nuclei (and indirectly the mammillary bodies) act upon the hippocampal formation rather than *vice versa*. This view can be contrasted with more traditional models (Papez, 1937; Delay & Brion, 1969; Aggleton & Brown, 1999) that have stressed the importance of the hippocampal inputs to the mammillary bodies and their relay to the anterior thalamic nuclei. To examine this alternative proposal, it is necessary first to determine whether it is anatomically plausible and then to assess whether the status of the hippocampus depends on these medial diencephalic inputs.

The anterior thalamic nuclei project directly to the hippocampal formation via the cingulum bundle. In monkeys, it appears that the anterior thalamic nuclei and the closely related lateral dorsal thalamic nucleus are one of the principal sources of thalamic projections to the hippocampal formation, with nucleus reuniens providing a further source of projections to the hippocampus (Amaral & Cowan, 1980; DeVito, 1980). The situation in the rat might be slightly different as, in this species, the hippocampal projections from the anterior thalamus are considerably outnumbered by those from the nonspecific midline nuclei, including nucleus reuniens (Wyss *et al.*, 1979; Vertes *et al.*, 2006). The specific sites of termination of the anterior thalamic projections within the primate hippocampal formation remain unknown, though in the rat these projections terminate across all subdivisions of the subicular complex (Shibata, 1993a). In addition, the anterior thalamic nuclei have an indirect route to the hippocampal formation via the retrosplenial cortex, a route which is reciprocal (Vann *et al.*, 2009a,b;). By these direct and indirect routes, there is considerable potential for the anterior thalamic nuclei to influence hippocampal function.

A small number of studies have made lesions in the anterior thalamic nuclei and examined markers of neuronal integrity in the hippocampus. Research using the immediate–early gene, c-*fos*, has revealed how both bilateral and unilateral anterior thalamic lesions are sufficient to reduce Fos levels in many hippocampal subfields (Jenkins *et al.*, 2002a,b;), especially in the dorsal (septal) hippocampus (see also Vann & Albasser, 2009). More diffuse medial diencephalic lesions, which include the anterior thalamic nuclei, midline thalamic nuclei and mammillary bodies, lead to depressed acetylcholine levels in the hippocampus and poor learning (Savage *et al.*, 2003; Roland & Savage, 2007). The principle that anterior thalamic activity might be vital for some aspect of hippocampal formation function was probably first demonstrated in the head-direction system, where it has been shown that the integrity of the anterior dorsal thalamic nucleus is necessary for the establishment of head-direction signals in the postsubiculum (Taube, 1998; Bassett & Taube, 2005). It is now becoming evident that the anterior thalamus may also be equally critical in a wider array of hippocampal activities.

As noted above, some of these anterior thalamic lesion effects upon the hippocampus may be via the loss of indirect, as well as direct, inputs. The potential contribution of the retrosplenial cortex stands out because, following anterior thalamic lesions, this cortical area displays the most dramatic depletions of immediate–early gene activity of any brain site so far examined (Aggleton, 2008). Anterior thalamic lesions almost completely eliminate Fos and zif268 production in layers II and upper III of the rat retrosplenial cortex, although the neurons appear grossly intact (Jenkins *et al.*, 2004; Poirier & Aggleton, 2009). Microarray studies have also shown that anterior thalamic lesions alter the activity of numerous other genes within the retrosplenial cortex, including those associated with metabolism (Poirier *et al.*, 2008). It is also the case that anterior thalamic lesions disrupt plasticity in slices of retrosplenial cortex tissue (Garden *et al.*, 2009). In view of the dense projections from the retrosplenial cortex to the hippocampal formation, there is clearly a strong likelihood that anterior thalamic pathology may additionally disrupt hippocampal processing via this indirect (retrosplenial) route.

This principle of indirect lesion effects was very clearly demonstrated by Vann & Albasser (2009), who compared the impact of mammillothalamic tract lesions and amygdala lesions on c-*fos* activity in a wide array of cortical and subcortical sites. While cutting the mammillothalamic tract caused retrograde degeneration in the mammillary bodies, it did not produce overt pathology in the anterior thalamic nuclei. Yet, despite the lack of any direct projections from the mammillary bodies to the hippocampus, mammillothalamic tract section significantly decreased Fos levels in the dorsal hippocampal formation as well as in the retrosplenial cortex and the prelimbic (medial prefrontal) cortex (Vann & Albasser, 2009). This study not only shows how widespread the neuronal dysfunctions might become after even a highly selective lesion within a medial diencephalic pathway but also reveals that many of these effects must be via indirect disconnections. These findings echo the results of PET studies in cases of patients with diencephalic amnesia, i.e., people typically with mammillary body or anterior thalamic pathology. Here, hypoactivity is consistently reported in the posterior cingulate–retrosplenial region (Fazio *et al.*, 1992; Joyce *et al.*, 1994; Paller *et al.*, 1997; Reed *et al.*, 2003) and is also often noted in the hippocampal region (Fazio *et al.*, 1992; Reed *et al.*, 2003). Likewise, an fMRI study of a man with nonalcoholic Wernicke–Korsakoff syndrome (i.e., mammillary body–anterior thalamic pathology) showed a lack of hippocampal recruitment in memory tasks that are typically associated with increased hippocampal activity (Caulo *et al.*, 2005). These hippocampal abnormalities may again reflect the complex combination of direct and indirect repercussions following anterior thalamic pathology.

The implication is that, after medial diencephalic pathology in the mammillary body → anterior thalamic axis, there are distal dysfunctions (both direct and indirect) in the retrosplenial cortex, parts of the prefrontal cortex, and the hippocampal formation (Vann & Albasser, 2009). Explaining why these distal changes might induce memory loss becomes an increasingly complex task, as it is necessary to consider the impact of dysfunctions in multiple regions rather than just understanding the roles of the direct projections back upon the temporal lobe. A further complexity is the strong likelihood of parallel mammillary → anterior thalamic nuclei pathways, each with different functions (see Fig. 5). There is, in addition, evidence that hippocampal damage will induce retrosplenial cortex dysfunction (Albasser *et al.*, 2007), i.e., the pattern of both direct and indirect influences is probably reciprocal.

The best described of the various parallel mammillary → anterior thalamic pathways (Fig. 5) involves the lateral mammillary bodies and the anterior dorsal nucleus. This pathway is used for the transmission of head-direction information (Vann & Aggleton, 2004), though even here it has often proved difficult to demonstrate a close coupling between activity in the head-direction system and spatial learning (Dudchenko *et al.*, 2005; Vann, 2005). One intriguing speculation relating to the head-direction system is that the cognitive processes that underlie the construction of scenes, mental imagery and mental navigation are all closely allied (Byrne *et al.*, 2007; Hassabis & Maguire, 2007), and that the above processes are lost in anterograde amnesia (Hassabis *et al.*, 2007; Bird & Burgess, 2008). It is then supposed that the head-direction system contributes to mental navigation and, in particular, to switching mental viewpoints. A more specific proposal is that the retrosplenial cortex has a key role in these processes as it is strategically placed to integrate interoceptive head-direction information (e.g., from the anterior dorsal thalamic nuclei) with exteroceptive information that uses both allocentric and egocentric frames of reference (Byrne *et al.*, 2007; Vann *et al.*, 2009a,b;). According to this view, the links that the retrosplenial cortex has with the anterior thalamic nuclei, the hippocampal formation, the prefrontal cortex and the parietal cortex would all be integral components supporting this complex cognitive task. While this account offers one possible line of explanation, it is most unlikely to be sufficient because the mammillary body–anterior thalamic axis has other nuclei (Fig. 5) that appear to contribute to learning but have quite different properties, e.g. cells showing theta rhythm (see Section 3). Therefore, the challenge is to assemble a multifactorial model that can explain the importance of the various projections from the anterior thalamus to the hippocampal formation, retrosplenial cortex and prefrontal cortex. This challenge is at two levels. The first is to explain how these regions interact to support memory in the intact brain. The second is to explain how medial diencephalic pathology can bring about a cascade of pathological changes that are sufficient to disrupt memory permanently.

We propose that each of the three major anterior thalamic nuclei comprises a key element in three distinct subsystems (with potentially a fourth system in the rat brain; see Fig. 5).

- Anterior medial ‘feed-forward’ system. The anterior medial nucleus receives inputs from the subiculum, dysgranular retrosplenial cortex and medial mammillary nucleus (more medial parts of the medial mammillary nucleus). Crucially, this nucleus has extensive reciprocal connections with a wide array of rostral cortical sites including the anterior cingulate and prelimbic cortices, but has only very limited projections back to the hippocampal formation (Wyss *et al.*, 1979; Shibata, 1993a,b; Shibata & Kato, 1993; Van Groen *et al.*, 1999). Only a small percentage of cells (6%) in this nucleus show rhythmic theta firing (Albo *et al.*, 2003). The prediction is that this system is primarily concerned with conveying integrated hippocampal–diencephalic signals to prefrontal mechanisms that aid cognitive flexibility, executive function and recency judgements. It is likely that one important element in this role will be to interact with the direct projections from the subiculum and field CA1 to the prelimbic cortex, i.e. bringing together the direct hippocampal → prefrontal connections with the indirect connections via the medial diencephalon.
- Anterior ventral ‘return loop’ system. The anterior ventral nucleus receives inputs from the subiculum, granular retrosplenial cortex and medial mammillary nucleus (more lateral parts of the medial mammillary nucleus). Unlike the anterior medial nucleus, the anterior ventral nucleus lacks, or has only very light, connections with rostral cortical sites but has widespread direct projections back to the subiculum, presubiculum and parasubiculum within the hippocampal formation (Wyss *et al.*, 1979; Shibata, 1993a,b; Van Groen & Wyss, 1995). This anterior thalamic nucleus also has more extensive projections to the retrosplenial cortex (an indirect route back to the hippocampus) than does the anterior medial nucleus. Recording studies indicate that 75% of cells in this nucleus fire rhythmically with theta (Albo *et al.*, 2003), strongly suggesting that a key functional element of this nucleus is the conveyance of theta to the hippocampal formation with its involvement in optimising synaptic plasticity (see Section 3).
- Anterior dorsal ‘head-direction’ system. The anterior dorsal nucleus receives inputs from the postsubiculum, the granular retrosplenial cortex and the lateral mammillary nucleus. The anterior dorsal nucleus projects to the postsubiculum and the granular retrosplenial cortex, but lacks frontal cortical connections (Wyss *et al.*, 1979; Shibata, 1993a,b; Van Groen & Wyss, 1995). Many of the cells in this nucleus, along with those in the lateral mammillary nucleus, have the highly distinctive electrophysiological property that they are tuned to a particular head direction but are insensitive to location, i.e. they behave rather like a compass (Taube, 1995). In contrast, only a small proportion of cells in this nucleus (12%) are thought to fire rhythmically with theta (Albo *et al.*, 2003). In rats, the head-direction system is assumed to support navigation while, in humans, it may have taken on additional roles in mental navigation and imagery manipulation (see above).

At present these three sets of different functions remain speculative, though they are all derived from anatomical and electrophysiological findings. Our goal has been to advance our understanding of the pathways that mediate hippocampal–anterior thalamic memory mechanisms, and to suggest a framework that may support future investigations. In doing so, it has also become apparent that there are multiple hippocampal–anterior thalamic pathways, each of which has a parallel, but different, organisation. Despite this multiplicity, it is assumed that all of these pathways contribute in some way to episodic memory function in humans. The task of understanding these functional relationships is made all the more difficult if one assumes that many of the hippocampal–thalamic interactions are reciprocal. Finally, many of the key interactions may prove to be indirect, e.g., via sites such as the retrosplenial cortex. This last consideration may have particular relevance for neurological conditions that induce pathology in specific sites within this extended hippocampal system, yet have much more distributed patterns of dysfunction.

At the start of this review, we stated that our goal was to explain five emergent concepts concerning hippocampal–anterior thalamic function. These concepts include the delineation of three parallel, but different, information streams within the anterior thalamic nuclei, allied to the notion that these medial diencephalic nuclei combine a mixture of properties, some of which depend on the hippocampus but, crucially, others of which are initially independent of the hippocampus. Emphasis has also been placed on trying to understand why these hippocampal–medial diencephalic interactions supporting memory should be reciprocal, and to determine for which forms of memory these interactions are most critical. Clinical findings now strongly indicate that, although these interactions are vital for episodic memory, they are not required for familiarity-based recognition. By considering data from a variety of disciplines, it has proved possible to expand on each of the above themes and to gain an insight into the potential complexities of the operations of these various pathways. Critical amongst these processes has been the specification of parallel hippocampal–diencephalic pathways, each one of which appears to support memory in different ways, yet they also appear to work together so that their joint functions become vital for our memory of day-to-day events.

## Abbreviations

BDNF: brain-derived neurotrophic factor
LTD: long-term depression
LTP: long-term potentiation
